# Telling the truth to patients and relatives

**DOI:** 10.4103/0019-5545.43628

**Published:** 2008

**Authors:** C. Shamasundar

**Affiliations:** 250, 43^rd^ Cross, 9^th^ Main, 5^th^ Block, Jayanagar, Bangalore - 560 041, India. E-mail: drshamasundar@yahoo.com

Sir,

The topic of ‘Doctor’ dilemma in truth telling' in the column “Periscope” of Indian Journal of Psychiatry,[1] was a timely reminder for serious debate on this important, but neglected component of clinical practice. I like to touch upon a few other aspects of this dilemma.

Opinion available in the literature ranges from a mandatory feedback of important clinical information to patient/family to withholding certain potentially traumatic information from the patient. The over-riding demand for absolute truth telling is in relation to informed consent.

‘Truth telling’ is a dilemma because of two reasons. First is that doctors are not formally trained in this task as a part of their routine undergraduate or postgraduate learning. And there are no well laid-out, generally agreed upon guidelines as to how to go about this task of ‘truth telling’.

Second reason is related to a certain degree of inherent uncertainty of our medical knowledge, especially in psychiatry. All medical knowledge especially psychosocial is statistical in nature with no certainties, but only differing degrees of probabilities. In such a scenario:

- The fact of certain degree of uncertainty itself becomes the truth.
- A serious question is: will this probability knowledge interfere with the patient's and family's hope, motivation, and determination to endeavour toward a positive outcome?
- Another question is: is it not our clinical duty to foster hope and confidence in patient and family
- If I myself were a patient, how would I want to reposition my hope, confidence, and determination *vis-à-vis* the uncertain outcome?

In this context, it is prudent to borrow three concepts from ancient Indian wisdom about ‘telling the truth’ that offer operational guidelines. These guidelines are scattered across the scriptures because ‘truthfulness’ is the highest virtue of human behavior. The first concept is ‘righteousness’. The second is ‘ability of an individual to understand and benefit from a given set of knowledge (truth)’. The third deals with ‘how to tell the truth?’ These three concepts can be extrapolated to the doctor-patient relationship.

## RIGHTEOUSNESS

General principles of righteousness are:

- In times of conflicts (dilemmas), choose that course of action which is righteous.
- In specific terms, righteousness consists of Universal compassion (Universal oneness), ensuring that anger, greed, and lust do not influence one's behavior, and behaving toward others as toward one's own self.[2]
- In more general terms, righteousness is a set of attitude and behavior that is conducive to most good in the long run to all life.
- What is righteous is dependent on time, place, person/s concerned, and circumstances. Thus, what is righteous in one situation need not be in another.
- These principles offer a framework within which decisions for further action can be taken based on other factors/variables instead of obsessional adherence to a stereotyped routine.

## ABILITY OF THE INDIVIDUAL TO UNDERSTAND AND BENEFIT FROM A GIVEN SET OF INFORMATION OR ‘KNOWLEDGE’

We, mental health professionals are all aware that the ability of people to understand the implications of information follows Guassian distribution like all natural phenomena. In the same way, their ability to benefit by such understanding too follows similar distribution. In such a situation how valid are the legal and ethical injunctions?

We also tend to overlook the fact that truth (facts of ‘reality’) is often very difficult to understand and very cruel in its effect. For example, imagine a patient recovering from ischemic heart disease hearing about his son's death in an accident, or about his comorbid cancer. Not everyone is capable of understanding a set of facts, assimilating their implications, and benefiting from them. People differ widely in this ability.

Ancient Indian wisdom stipulates that there is such a thing as an individual’ ability or lack of it to understand and benefit from a given knowledge. Sometimes, the ‘knowledge’ can be misunderstood and misused to a dangerous extent. This negative potential is best illustrated in a Sufi story.[3]

## HOW TO IMPART INFORMATION TO THE PATIENT/FAMILY

This issue involves such considerations as: what to tell, how much to tell, how to tell, and whom to tell (e.g., to a chosen family member instead of the patient).

Some of these aspects are explained as components of ideal communication by a lady sage.[4] Among other requirements, the listener must be eager to learn, speech must be capable of being understood by the listener, and the message must be useful to the listener.

It may even become necessary at times for the patient or family to be trained or prepared in a step-ladder fashion to become able to assimilate the information intended to be imparted.

During my undergraduate training (1950s), we were taught: “…best surgeon is he who knows when not to operate...best physician is he who knows his limitations…” I believe that this wisdom still holds true. In a similar fashion, I would say “best psychiatrist is he who knows what to say, what not to say, when to say, how to say, and whom to say.” The foundations for the doctor to learn this art must be laid in undergraduate and postgraduate training.
